# A Sensitive Secondary Users Selection Algorithm for Cognitive Radio *Ad Hoc* Networks

**DOI:** 10.3390/s16040445

**Published:** 2016-03-26

**Authors:** Aohan Li, Guangjie Han, Liangtian Wan, Lei Shu

**Affiliations:** 1 Department of Information and Communication System, Hohai University, Changzhou 213022, China; liaohan1989@gmail.com (A.L.); wanliangtian1@163.com (L.W.); 2 Department of Signal and Information Processing, Heilongjiang Univetsity, Harbin 15006, China; 3 Guangdong Petrochemical Equipment Fault Diagnosis Key Laboratory, Guangdong University of Petrochemical Technology, Maoming 525000, China; lei.shu@ieee.org

**Keywords:** SSUS, CRAHNs, network architecture, energy efficiency, real-time

## Abstract

Secondary Users (SUs) are allowed to use the temporarily unused licensed spectrum without disturbing Primary Users (PUs) in Cognitive Radio *Ad Hoc* Networks (CRAHNs). Existing architectures for CRAHNs impose energy-consuming Cognitive Radios (CRs) on SUs. However, the advanced CRs will increase energy cost for their cognitive functionalities, which is undesirable for the battery powered devices. A new architecture referred to as spectral Requirement-based CRAHN (RCRAHN) is proposed to enhance energy efficiency for CRAHNs in this paper. In RCRAHNs, only parts of SUs are equipped with CRs. SUs equipped with CRs are referred to as Cognitive Radio Users (CRUs). To further enhance energy efficiency of CRAHNs, we aim to select minimum CRUs to sense available spectrum. A non-linear programming problem is mathematically formulated under the constraints of energy efficiency and real-time. Considering the NP-hardness of the problem, a framework of a heuristic algorithm referred to as Sensitive Secondary Users Selection (SSUS) was designed to compute the near-optimal solutions. The simulation results demonstrate that SSUS not only improves the energy efficiency, but also achieves satisfied performances in end-to-end delay and communication reliability.

## 1. Introduction

The concept of Cognitive Radio (CR) technology is proposed to address the problems in wireless networks resulting from the limited available spectrum and the inefficiency in the spectrum usage [1,2,3]. CR is the key enabling technology that enables SUs to exploit the licensed spectrum opportunistically without interfering with PUs [4,5,6]. According to the network architecture, Cognitive Radio Networks (CRNs) can be classified as the CRAHNs and the infrastructure-based CRNs. In infrastructure-based CRNs, the observations and analysis performed by each CR user feed the central CR based-station. On the contrary, each user needs to have all CR capabilities and is responsible for determining its actions based on the local observation in CRAHNs [7,8,9,10,11]. We focus on the CRAHNs in this paper.

Existing architectures for CRAHNs assume that all SUs are equipped with CRs and sense available spectrum by themselves [12,13,14]. However, a large amount of time and effort have to be spent on development of CR [15,16,17,18]. In addition, there is no reason to enforce SUs to replace their device. Furthermore, CR will increase energy cost for its cognitive functionality [19,20,21,22,23]. The energy cost for cognitive functionality is undesirable for the battery powered devices [24,25,26,27,28,29,30,31].

**Figure 1:**
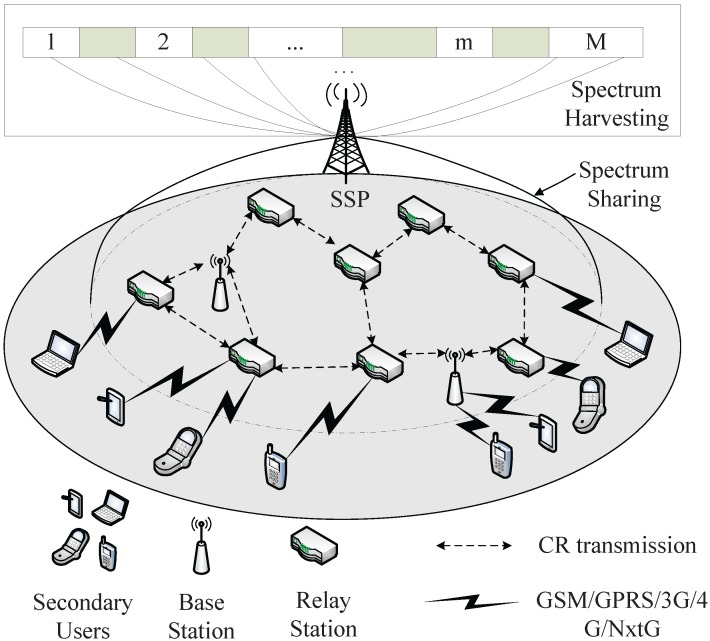
System architecture of CCH.

The above issues have drawn some attention from the research community. In [32,33], a network architecture, referred to as Cognitive Capacity Harvesting network (CCH), is proposed to enhance the spectrum and energy efficiencies of CRNs. Figure 1 shows the system architecture of the CCH. CCH consists of four entities: a Secondary Service Provider (SSP), Base Stations (BSs), Relay Stations (RSs) and SUs. Both BSs and RSs are equipped with CRs, which can use idle licensed spectrum for communication without disturbing PUs. There is no specific requirement on CR imposed on SUs. If SUs do not have the capability of cognition, BSs and RSs can switch to the unlicensed spectrum to provide communication services. If SUs have cognitive capability, they can communicate with BSs and RSs through both licensed and unlicensed spectrum. BSs and RSs collect the service demands from SUs as well as statics for spectrum availability. Then, BSs and RSs submit them to SSP through the common control channels. SSP is an independent wireless service. Under the guidance of SSP, SUs access their nearby BSs and RSs and deliver packets via BSs and RSs using both licensed and unlicensed spectrum. CCH addresses the disadvantages of traditional CRN architectures. However, extra unnecessary infrastructures are brought to CRNs, which unavoidably add too much cost and complexity to CRNs.

Considering the existing problems of CRN architecture mentioned above, a new architecture referred to as RCRAHN is proposed in this paper. RCRAHN is based on the spectral requirements of SUs. In RCRAHNs, there is no specific requirement on CRs imposed on SUs. SUs can make a tradeoff among their spectral requirement, equipment cost and energy consumption. Then, they make a decision about whether to equip with CR or not. In this paper, SUs can be classified into CRUs and Non-Cognitive Radio Users (NCRUs). If the SUs are equipped with CRs, they are referred to as CRUs. CRUs can opportunistically use the vacant licensed spectrum. If the SUs are not equipped with any CRs, they are referred to as NCRUs. NCRUs cannot use licensed spectrum. CRUs can also make a tradeoff between their spectral requirement and energy consumption. Then, a decision about whether to open CR or not will be made. If CRUs open their CR, they are referred to as Opened Cognitive Radio Users (OCRUs). Otherwise, they are referred to as Closed Cognitive Radio Users (CCRUs). To further enhance energy efficiency, we aim to select minimum OCRUs to sense available licensed spectrum. OCRUs, which are selected to sense available licensed spectrum, are referred to as Sensible Secondary Users (SSUs). Otherwise, they are referred to as Non-Sensible Secondary Users (NSSUs).

RCRAHN can address the concerns that we have mentioned above. In RCRAHNs, SUs can make a decision about whether to equip with CRs. In addition, CRUs can make a decision about whether to open CRs. Furthermore, only parts of OCRUs are selected as SSUs. Compared with traditional CRAHNs, RCRAHNs will not impose CRs on SUs while meeting the communication requirements of SUs. Compared with CCH, RCRAHNs will not bring extra facility to CRNs. Therefore, RCRAHNs successfully exploit CR technology while reducing the complexity of SUs. In addition, spectral requirement, equipment cost and energy consumption are considered in RCRAHNs, which can further optimize the performances of CRAHNs.

In this paper, we study the selection strategy of SSUs. Energy efficiency and real-time are considered when the selection strategy is designed. Energy efficiency refers to ensuring that all OCRUs in RCRAHNs can achieve their available license spectrum information with maximum power. Real-time refers to ensuring that the available licensed spectrum information can be transmitted to NSSUs during its lifetime. Lifetime of the information can be interpreted as follows. If the duration after information generation does not exceed the lifetime of the information, information is effective. Otherwise, information is invalid. The main contributions of our paper can be summarized as follows:

- A new architecture referred to as RCRAHN is proposed, which takes the spectral requirement, equipment cost and energy consumption into account.
- Based on RCRAHN, we address the problem of minimizing the number of SSUs. We transform this problem into a non-linear programming problem. Energy efficiency and real-time constraints are considered.
- The non-linear programming problem that we mathematically formulated is an NP-hardness problem. Hence, in order to solve the minimum SSU selection problem, a framework of a heuristic algorithm, referred to as SSUS, was designed.

The rest of this paper list organized as follows. In Section 2, we introduce the system model and describe the problem formulation. The non-linear programming problem is mathematically formulated under the energy efficiency and real-time constraints in Section 3. The heuristic algorithm SSUS is presented in Section 4. SSUS is validated in Section 5, followed by the conclusions in Section 6.

## 2. System Model and Problem Formulation

### 2.1. System Model

A novel architecture called RCRAHN is proposed in this paper. In RCRAHNs, SUs can make a decision about whether to equip CRs or not according to their spectral requirement, equipment cost and energy consumption. The status of CRs can be classified into open status and closed status in RCRAHNs. Open status can be further classified into sensible status and insensible status.

In RCRAHNs, SUs equipped with CRs are referred to as CRUs. Otherwise, they are referred to as NCRUs. CRUs whose CRs are under the closed status are referred to as CCRUs. NCRUs and CCRUs cannot use spectrum in a dynamic manner. CRUs whose CRs are under the open status are referred to as OCRUs. OCRUs can use spectrum in a dynamic manner. If the CRs of OCRUs are under the sensible status, they are called SSUs. Otherwise, they are called NSSUs. SSUs and NSSUs can use spectrum in a dynamic manner. SSUs can capture or sense the information from its radio environment, while NSSUs do not have this ability. Table 1 gives the description of different types of SUs.

**Figure 2:**
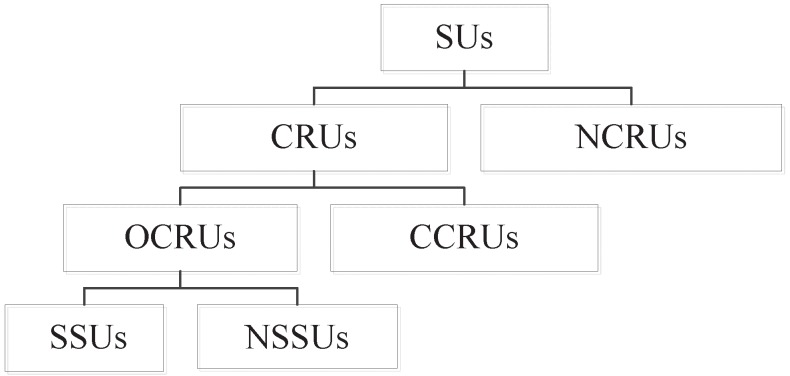
The relationship between different types of SUs.

The relationship between different types of SUs can be summarized as follows: SUs are classified into CRUs and NCRUs. CRUs are classified into OCRUs and CCRUs. OCRUs are classified into SSUs and NSSUs. Figure 2 shows the relationship between different types of SUs in RCRAHNs.

**Figure 3:**
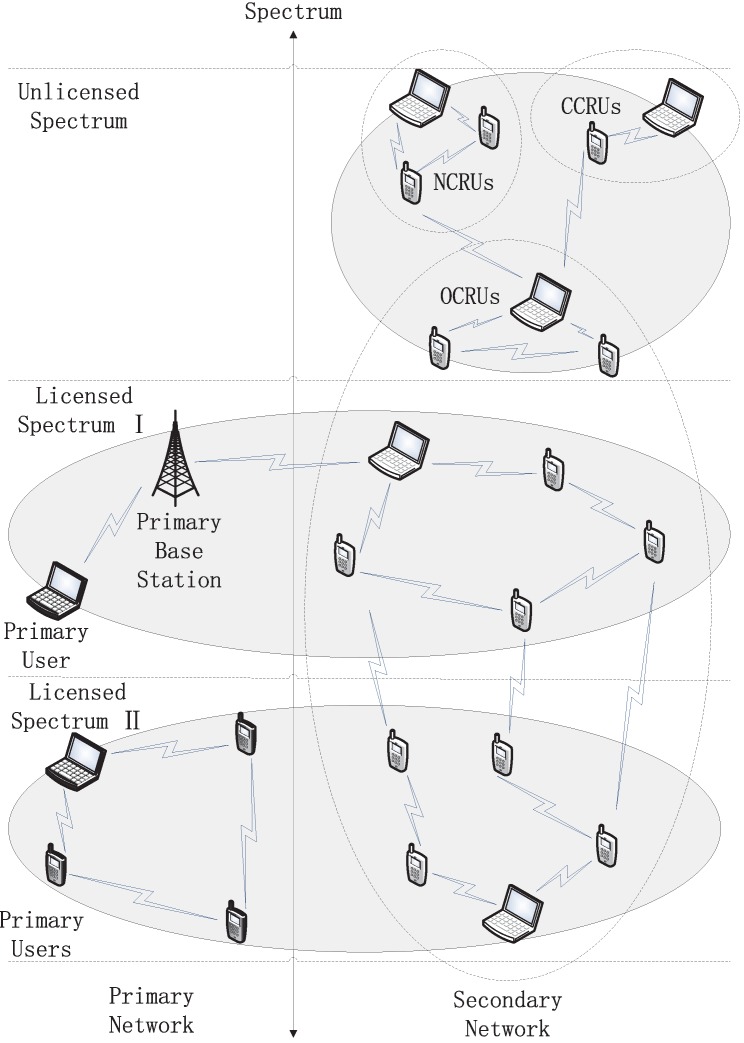
System Architecture of RCRAHNs.

Figure 3 shows the system architecture of RCRAHNs. As shown in Figure 3, SUs mainly consist of three types, which are OCRUs, CCRUs and NCRUs, respectively. SSUs and NSSUs are included in OCRUs. Both NCRUs and CCRUs are only allowed to use unlicensed spectrum. OCRUs are allowed to use both unlicensed and licensed spectrum. PUs still use licensed spectrum to transmit data.

Consider an RCRAHN with |N| OCRUs at time *t*. We denote *N* as the set of OCRUs. Only OCRUs can use spectrum in a dynamic manner in RCRAHNs. When OCRUs transmit data with PUs simultaneously, interference temperature limit should not be violated. Hence, OCRUs have to accurately identify the available spectrum. Energy of OCRUs will be consumed due to sensing and gathering information from their surrounding environment. Therefore, to enhance energy efficiency, we aim to select |n| SSUs among |N| OCRUs to sense and gather available spectrum information. We denote *n* as the set of SSUs, where n⊆N. Let |N| denote the number of OCRUs in RCRAHN. Let |n| denote the number of SSUs in RCRAHN, and |n|≤|N|. To guarantee the Quality of Service (QoS) of communication, |n| SSUs have to accurately sense and gather information for |N| OCRUs. In this paper, we aim to address the problem of minimizing the number of SSUs based on RCRAHN. The minimum SSUs have to sense and gather the available spectrum information for all OCRUs accurately.

The communication radius of PUs is represented by *R*. Assume the OCRUs within the communication radius of PUs are not allowed to use licensed spectrum when the licensed spectrum is occupied by PUs. *R* is related to the maximum transmission power at sender.

In RCRAHN, we define the neighboring area of OCRUs as their surrounding regions. If two OCRUs are in a mutual neighboring area, they can communicate with each other. Assume all OCRUs use the same accessing technology in RCRAHN. Assume the maximum transmission power of all OCRUs is identical while the maximum transmission range of all OCRUs is identical as well in RCRAHN. In addition, we assume that the coverage area of the neighboring area is identical for OCRUs. Coverage area can be interpreted as follows. When OCRU uses the maximum transmission power to communication, the area where the communication radius of OCRU covers is called coverage area. Let *r* denote the communication radius of OCRUs. Hence, the communication radius of OCRU *m* can be represented by $r_{m}$. The neighboring area of OCRU *m* contains the coverage area of its communication radius. Note that r<<R. Therefore, we can assume that OCRUs in mutual neighboring areas are influenced by the same PUs, which means that OCRUs in mutual neighboring areas have the same available licensed spectrum at the same time. OCRUs in a neighboring area of OCRU *m* can consider the available licensed spectrum information sensed by OCRU *m* as their available spectrum information. The minimum OCRU selection problem can be addressed based on the aforementioned description.

**Figure 4:**
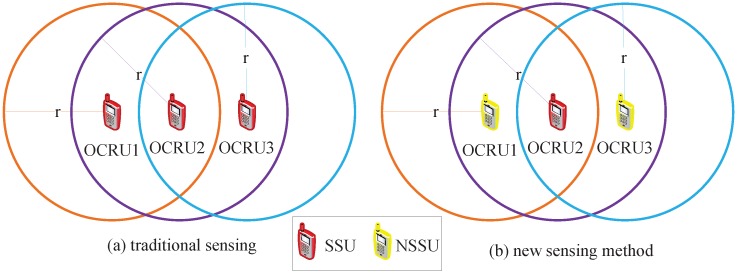
Sensing model. (**a**) traditional sensing, (**b**) new sensing method.

Sensing mode of the traditional CRAHNs and the RCRAHNs are illustrated in Figure 4. OCRU1 and OCRU2 are included in the neighboring area of OCRU2 as shown in Figure 4. In traditional CRAHNs, OCRU1, OCRU2 and OCRU3 need to sense and gather available licensed spectrum information as shown in Figure 4a. In RCRAHNs, OCRU2 can sense and gather available licensed spectrum information for OCRU1 and OCRU3 as shown in Figure 4b. OCRUs in the neighboring area of OCRU2 can get their available licensed spectrum from OCRU2. Hence, only OCRU2 senses and gathers information among OCRU1, OCRU2 and OCRU3. The minimum SSU selection problem is formulated based on the description mentioned above.

### 2.2. Problem Formulation

In this paper, we consider the selection problem of minimum SSUs. In other words, we aim to select minimum SSUs among |N| OCRUs. The minimum SSUs have to ensure that all OCRUs in RCRAHNs can achieve their available licensed spectrum information. The problem can be formulated as a non-linear programming problem under the energy efficiency and real-time constraints [34]. Energy efficiency refers to ensuring that all OCRUs in RCRAHNs can achieve their available licensed spectrum information with maximum power. Real-time refers to ensuring that the available licensed spectrum information can be transmitted to NSSUs during the lifetime of the information. Energy efficiency and real-time constraints can be summarized as Condition 1 and Condition 2, respectively.

Condition 1: OCRUs are movable in RCRAHNs. To guarantee that every OCRU can achieve its available spectrum information, at least one of the same SSUs has to exist in the neighboring area of one NSSU during every spectrum sensing and information transmission time.

Condition 2: It should be ensured that the available spectrum information sensed by SSUs can be transmitted to the NSSUs in their neighboring areas during the lifetime of the information.

## 3. Optimal SSU Selection

In this section, spectrum efficiency and real-time constraints are mathematically expressed. Furthermore, the minimum SSU selection problem is is formulated into a non-linear programming problem [35].

### 3.1. Communication Range

Suppose all OCRUs in RCRAHN use the same power for transmitting. Consider the following power propagation model [36]

(1) $$P_{r}=\gamma d^{-\alpha }P_{t}$$

where *γ* is an antenna-related constant, *d* is the distance between transmitter and reviver, and *α* is the pass loss factor. $P_{t}$ and $P_{r}$ denote the transmission power at sender and the received power at receiver, respectively. We assume that communication can succeed only when the power received at the OCRU exceeds $P_{k}$. Let *P* denote the maximum transmission power of OCRUs. Then, when OCRUs are able to communicate with each other, they have to satisfy

(2) $$P_{k}\leq \gamma d^{-\alpha }P$$

Hence, OCRUs can communicate with each other when the distance between them satisfies

(3) $$d\leq {\left(\gamma P/P_{k}\right)}^{{}^{1}/{}_{\alpha }}$$

Equation (3) shows that the communication between two OCRUs can be successful when the distance between them is no more than ${\left(\gamma P/P_{k}\right)}^{{}^{1}/{}_{\alpha }}$. We define ${\left(\gamma P/P_{k}\right)}^{{}^{1}/{}_{\alpha }}$ as the communication range of OCRUs, which is denoted as *r*. Hence, it should be satisfied that the distance between SSUs and NSSUs is no more than *r* to ensure the available spectrum information sensed by SSUs can be successfully transmitted to NSSUs Available spectrum information sensed by SSUs can be transmitted to NSSUs by using the common channels between them, *i.e.*, if the distance between NSSU *o* and SSU *m* is no more than *r*, the available spectrum information sensed by SSU *m* can be considered as the available spectrum information of NSSU *o*.

### 3.2. Objective Function

Our objective is to minimize the number of SSUs in RCRAHNs under the energy efficiency and real-time constraints, which can be expressed as

(4) $$\min_{n\subseteq N}\;\left|n\right|$$

### 3.3. Energy Efficiency Constraint

We define a time $T_{s}$ as each sensing time of SSUs. Let $T_{t}$ be the lifetime of the available spectrum information. Hence, available spectrum information sensed by SSUs have to be transmitted to NSSUs in their neighboring areas during $T_{t}$. NSSUs can accurately achieve their available licensed spectrum information only when at least one of the same SSUs exists in its neighboring area during $T_{s}+T_{t}$. Let $\delta_{si}\left(t\right)$ be a binary, and it stands for denoting the communication relationship between SSU *i* and NSSU *s*. If $d_{si}\leq r$ during $\left[t_{i}^{+},t_{i}^{+}+T_{s}+T_{t}\right]$, $\delta_{si}\left(t\right)=1$. Otherwise, $\delta_{si}\left(t\right)=0$. This can be expressed as

(5) $$\delta_{si}\left(t\right)=\left\{\begin{array}{cc} 1, & \mathrm{if}d_{si}\leq r\mathrm{during}\left[t_{i}^{+},t_{i}^{+}+T_{s}+T_{t}\right]\\ 0, & \mathrm{otherwise} \end{array}\right.$$

where $d_{si}$ is the distance between SSU *i* and NSSU *s*. SSU *i* starts to sense available licensed spectrum information at $t_{i}^{+}$. Note that when $\delta_{si}\left(t\right)=1$, SSU *i* can maintain communication with NSSU *s* during $\left[t_{i}^{+},t_{i}^{+}+T_{s}+T_{t}\right]$.

Let $\Pi_{s}$ be a binary variable denoting whether OCRU *s* is an SSU. If OCRU *s* is an SSU, $\Pi_{s}$ = 1. Otherwise, $\Pi_{s}$ = 0, *i.e.*,

(6) $$\Pi_{s}=\left\{\begin{array}{cc} 1, & \text{if OCRU}s\text{ is a SSU}\\ 0, & \mathrm{otherwise} \end{array}\right.$$

According to the energy efficiency constraint, OCRU in RCRAHN has to satisfy that it is an SSU, or it is an NSSU when at least one of the same SSUs exists in its neighboring area during $\left[t_{i}^{+},t_{i}^{+}+T_{s}+T_{t}\right]$. Hence, the optimal SSU selection problem has to satisfy the following energy efficiency constraint:

(7) $$1-\prod_{OCRU\;i\;\in \;n}\left(1-\Pi_{s}\cdot \delta_{si}\left(t\right)\right)\cdot \left(1-\Pi_{s}\right)>0$$

### 3.4. Real-Time Constraints

According to the Shannon–Hartley theorem, when SSU *i* transmits data to NSSU *j*, the capacity of link (*i,j*) can be calculated as

(8) $$C_{ij}=W_{ij}\log_{2}\left(1+\frac{\gamma d_{ij}^{-\alpha }P_{ij}}{N_{0}}\right)$$

where $W_{ij}$ is the communication bandwidth between SSU *i* and NSSU *j*. $P_{ij}$ is the transmission power of SSU *i* when it transmits data to NSSU *j*. $N_{0}$ is the noise power.

We assume that SSU *i* senses available licensed spectrum information for *m* NSSUs. Let $S_{\begin{array}{c} i \end{array}}$ be the sensing set of SSU *i*, which is formed by *m* NSSUs. Let $|S_{i}|$ be the number of NSSUs in $S_{\begin{array}{c} i \end{array}}$, and $|S_{i}|=m$.

The Broadcast way is used by SSU *i* to transmit its sensed information to the NSSUs in its sensing set $S_{\begin{array}{c} i \end{array}}$. Let *w* be the size of the available licensed spectrum information sensed by SSU *i*. It will spend $\frac{w}{C_{ij}}$ time for SSU *i* to transmit its sensed information to $\forall NSSU\;j\in S_{i}$. Therefore, the time used by SSU *i* to transmit its sensed information to all NSSUs in $S_{\begin{array}{c} i \end{array}}$ can be calculated as

(9) $$\frac{w}{\min_{NSSU\;j\;\in \;S_{i}}\;C_{ij}}$$

The available licensed spectrum information of OCRUs is time-varying. Hence, to ensure the real-time of the sensed information, the information sensed by SSU *i* should be transmitted to all NSSUs in $S_{\begin{array}{c} i \end{array}}$ during the lifetime of the information. Denote the lifetime of the sensed information as $t_{\delta }$. If the transmission time of the sensed information exceeds $t_{\delta }$, the sensed information will be aborted. Therefore, the following constraint should be satisfied for ∀SSUi∈n

(10) $$\frac{w}{\min_{NSSU\;j\;\in \;S_{i}}\;C_{ij}}<t_{\delta }$$

where $t_{\delta }$ is the maximum transmission delay of the available licensed spectrum information. $t_{\delta }$ is referred to as the maximum tolerable delay. By substituting Equation (8) into Equation (10), we are able to transform Equation (10) into the following constraint

(11) $$\frac{w}{\min_{NSSU\;j\;\in \;S_{i}}\;\left[W_{ij}\log_{2}\left(1+\frac{\gamma d_{ij}^{-\alpha }P_{ij}}{N_{0}}\right)\right]}<t_{\delta }$$

By integrating the objective function with the energy efficiency and real-time constraints, the minimum SSU selection problem based on RCRAHNs can be formulated as follows:

$$\begin{array}{cc} & \;\;\;\;\;\;\;\;\;\min_{n\subseteq N}\;\left|n\right|\\ & s.t.\;\;1-\prod_{OCRU\;i\;\in \;n}\left(1-\Pi_{s}\cdot \delta_{si}\left(t\right)\right)\cdot \left(1-\Pi_{s}\right)>0\;\\ & \;\;\;\;\;\;\;\;\;\;\;\;\;\;\;\;(\forall \mathrm{OCR}U\;s\;\in \;N)\;\;\;\;\;\;\\ & \;\;\frac{w}{\min_{NSSU\;j\;\in \;S_{i}}\;\left[W_{ij}\log_{2}\left(1+\frac{\gamma d_{ij}^{-\alpha }P_{ij}}{N_{0}}\right)\right]}<t_{\delta }\;\\ & \;\;\;\;\;\;\;\;(\forall OCRU\;i\;\in \;n,\;\forall NSSU\;j\;\in \;S_{i})\;\;\\ & \;\; \end{array}$$

## 4. Heuristic Algorithms

### 4.1. Computational Complexity

The non-linear programming problem that we constructed is a set cover problem [37,38]. The set cover problem has been proved to be an NP-hardness problem [39,40]. Therefore, the non-linear programming problem that we constructed is also an NP-hardness problem. In this section, we develop an efficient heuristic algorithm referred to as SSUS for the minimum SSU selection problem to find the near-optimal solutions (Algorithm 1).

### 4.2. Framework of the SSUS

Each OCRU in RCRAHNs calculates the number of OCRUs in its neighboring area. Denote the number of OCRUs as communication number. *i.e.*, if there are *f* OCRUs in the neighboring area of one OCRU, the communication number of this OCRU is *f*. For one OCRU, denote the set *X* as its communication set, which consists of itself and OCRUs in its neighboring area. Then, OCRUs in *X* are sorted in a descending order according to their communication number to form a new set $Y=\{y_{1},y_{2},\cdot \cdot \cdot ,y_{M}\}$. Note that if OCRUs have the same communication range, only the OCRU which has the minimum *T* participates in sorting. In addition, if OCRUs in *X* have the same communication number, they will be ordered as *T*. Then, the OCRU which has the minimum *T* is reserved, and other OCRUs which have the same communication number with this OCRU are deleted. The $T_{i}$ of OCRU *i* can be calculated as

(12) $$T_{i}=\frac{w}{\min_{OCRU\;i\;\in \;X_{i}}\;\left[W_{ij}\log_{2}\left(1+\frac{\gamma d_{ij}^{-\alpha }P_{ij}}{N_{0}}\right)\right]}$$

Then, the verification procedure is conducted. The verification procedure starts at the first OCRU in set *Y*. OCRUs are verified as to whether they can satisfy $T<t_{\delta }$. If OCRU *i* satisfy $T_{i}<t_{\delta }$, OCRU *i* will be selected as an SSU, then the verification procedure is finished. OCRUs in the neighboring area of OCRU *i* can achieve their available licensed spectrum information from OCRU *i*. If OCRU *i* does not satisfy $T_{i}<t_{\delta }$, the next OCRU in set *Y* will be verified. Note that if all OCRUs in set *Y* are verified, and none of the OCRUs satisfy $T<t_{\delta }$, then OCRU itself will be selected as an SSU.

**Algorithm 1** SSUS1:Each OCRU calculates its communication number;2:Each OCRU and OCRUs in their neighboring areas form a set *X*;3:OCRUs in *X* are sorted in a descending order according to their communication number and form a new set $Y=\{y_{1},y_{2},\cdot \cdot \cdot ,y_{M}\}$;4:**for** k←1 to M-1 **do**5:  Compute the value of $T_{k}$ by using Equation (12);6:  Compare $T_{k}$ with $t_{\delta }$;7:  **if** $T<t_{\delta }$ **then**8:    Stop at the current step and $y_{k}$ will be selected as a SSU;9:**  else**10:    Proceed to observe the next OCRU in *Y*;11:**  end if**12:** end for**13:OCRU itself will be selected as a SSU.

## 5. Simulation and Analysis

In this section, we illustrate the performance of the SSUS based on RCRAHNs by simulation. Assume that all SUs and PUs are randomly located in a square area. Assume that the number of SUs, CRUs and OCRUs are 100, 80 and 60, respectively. The communication range of PUs is set to be 60 m. We adopt a random waypoint model to model the motion of PUs and SUs. A fading channel model with Additive White Gaussian Noise (AWGN) is considered.

For evaluating the proposed algorithm, our simulations are based on the comparison with another two algorithms: (1) Random selection algorithm based on RCRAHNs; (2) Random selection algorithm based on the traditional CRAHNs. A random selection algorithm randomly selects one SSU from the OCRUs each time under the energy efficiency and real-time constraints. The influence of network size is also considered in simulation. We investigate the performance of SSUS based on the following performance metrics:

- The relationship between the maximum tolerable delay $t_{\delta }$ and the number of SSUs.
- The relationship between the communication range of SUs and the number of SSUs.
- The relationship between the simulation time and the average energy consumption.
- The relationship between the maximum tolerable delay $t_{\delta }$ and the packet loss rate.

**Figure 5:**
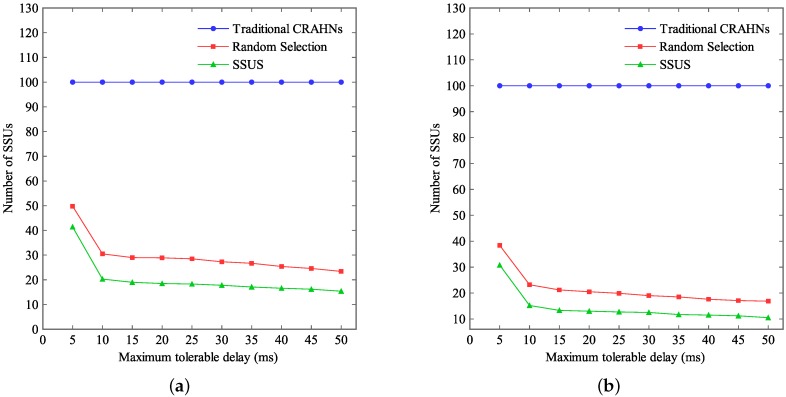
Number of SSUs *vs.* $t_{\delta }$. (**a**) Network size is 100×100 m${}^{2}$, (**b**) Network size is 60×60 m${}^{2}$.

Figure 5 reports the number of SSUs when the maximum tolerable delay $t_{\delta }$ varies. In this study, the communication range of SUs is fixed at 15 m. The network size is fixed at 100×100 m${}^{2}$ and 60×60 m${}^{2}$, respectively. Simulation results in Figure 5a are simulated under the network size of 100×100 m${}^{2}$. Simulation results in Figure 5b are simulated under the network size of 60×60 m${}^{2}$. $t_{\delta }$ varies from 0 ms to 50 ms.

As shown in Figure 5, we notice that the number of SSUs does not vary with $t_{\delta }$. Furthermore, the number of SSUs equals the number of SUs in traditional CRAHNs. The reason is that all SUs sense available spectrum information in traditional CRAHNs. However, the number of SSUs increases when the maximum tolerable delay $t_{\delta }$ goes up in RCRAHNs. The number of SSUs attained by SSUS is no more than that attained by random selection algorithm in RCRAHNs. Therefore, SSUS based on RCRAHNs can enhance energy efficiency compared with another two algorithms. In addition, the number of SSUs decreases under the same $t_{\delta }$ when the network size goes down in RCRAHNs.

**Figure 6:**
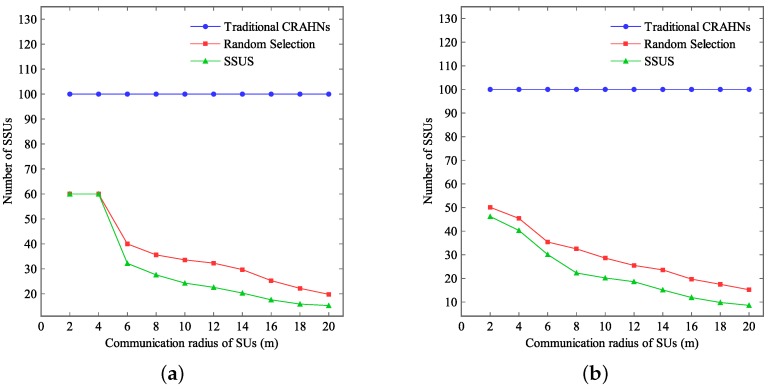
Number of SSUs *vs.* communication radius of SUs. (**a**) Network size is 100×100 m${}^{2}$, (**b**) Network size is 60×60 m${}^{2}$.

Figure 6 presents the number of SSUs when the communication range (*i.e.*, communication radius) of SUs varies. In this study, the maximum tolerable delay $t_{\delta }$ is fixed at 25 ms. The network size is fixed at 100×100 m${}^{2}$ and 60×60 m${}^{2}$, respectively. Simulation results in Figure 6a are simulated under the network size of 100×100 m${}^{2}$. Simulation results in Figure 6b are simulated under the network size of 60×60 m${}^{2}$. The communication range of SUs varies from 0 m to 20 m.

From Figure 6, it can be observed that the number of SSUs decreases when the communication range of SUs goes up in RCRAHNs. The reason is that more OCRUs will in the neighboring area of SSUs when the communication range of SUs goes up. Hence, more OCRUs can obtain their available licensed spectrum from one SSU when the communication range of SUs goes up in RCRAHNs. The number of SSUs attained by SSUS is no more than that attained by random selection algorithm in RCRAHNs, depending on the communication range of SUs. Thus, SSUS based on RCRAHNs can enhance energy efficiency compared with another two algorithms. Furthermore, it can be observed that the number of SSUs decreases with the decrease of the network size under the same situation.

**Figure 7:**
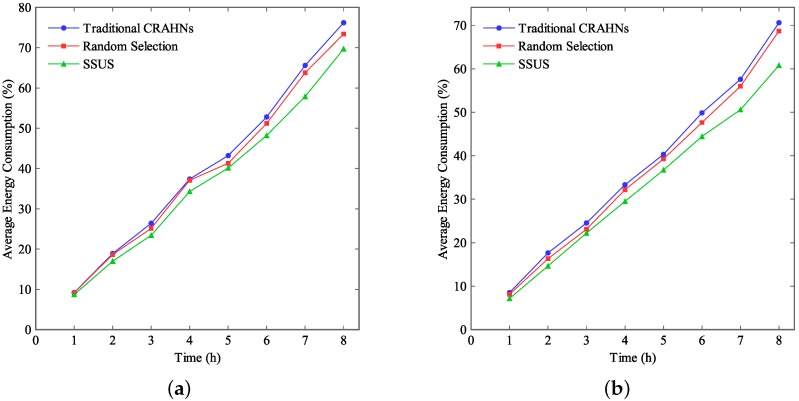
Average energy consumption *vs.* time. (**a**) Network size is 100×100 m${}^{2}$, (**b**) Network size is 60×60 m${}^{2}$.

Figure 7 demonstrates the average energy consumption when the simulation time varies. Define the average energy consumption as the ratio between the energy consumption of all SUs and the total energy of all SUs. In this study, the maximum tolerable delay $t_{\delta }$ and the communication range of SUs are fixed at 25 ms and 15 m, respectively. The network size is fixed at 100×100 m${}^{2}$ and 60×60 m${}^{2}$, respectively. Simulation results in Figure 7a are simulated under the network size of 100×100 m${}^{2}$. Simulation results in Figure 7b are simulated under the network size of 60×60 m${}^{2}$.

From Figure 7, it can be seen that the average energy consumption increases when the simulation time goes up both in traditional CRAHNs and RCRAHNs. In addition, the average energy consumption attained by SSUS in RCRAHNs is less than that attained by random selection in RCRAHNs and traditional CRAHNs. Therefore, SSUS based on RCRAHNs can enhance energy efficiency compared with another two algorithms. Furthermore, it can be observed that the average energy consumption decreases when the network size goes down at the same simulation time.

**Figure 8:**
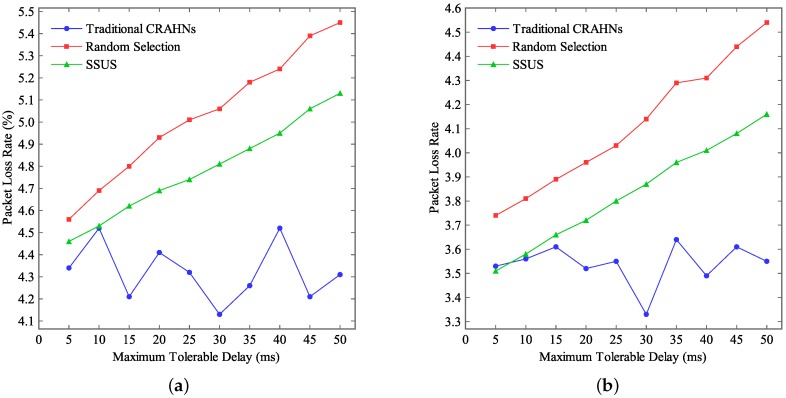
Packet loss rate *vs.* $t_{\delta }$. (**a**) Network size is 100×100 m${}^{2}$, (**b**) Network size is 60×60 m${}^{2}$.

Figure 8 shows the packet loss rate when the maximum tolerable delay $t_{\delta }$ varies. Define the packet loss rate as the ratio between the number of the lost packets and the total packets. In this study, the communication range of SUs is fixed at 15 m. The network size is fixed at 100×100 m${}^{2}$ and 60×60 m${}^{2}$, respectively. Simulation results in Figure 8a are simulated under the network size of 100×100 m${}^{2}$. Simulation results in Figure 8b are simulated under the network size of 60×60 m${}^{2}$. The maximum tolerable delay $t_{\delta }$ varies from 0 ms to 50 ms.

As shown in Figure 8, it can be seen that the packet loss rate attained by the random selection algorithm in traditional CRAHNs almost does not vary with $t_{\delta }$. The packet loss rate attained by SSUS and the random selection algorithm in RCRAHNs increases when $t_{\delta }$ goes up. Furthermore, the packet loss rate attained by random selection algorithm in traditional CRAHNs is always lower than that attained by SSUS and random selection algorithm in RCRAHNs. However, the difference is not too obvious. In addition, the packet loss rate attained by SSUS is always lower than that attained by random selection algorithm in RCRAHNs. Furthermore, it can be observed that the packet loss rate increases when the network size goes up.

In summary, numerical results demonstrate that the proposed SSUS based on RCRAHNs can enhance the energy efficiency. Meanwhile, it can meet the requirements of the end-to-end delay and communication reliability.

## 6. Conclusions

In this paper, we first constructed a new architecture named RCRAHNs, in which SUs can make a decision about whether to equip CRs or not according to their spectral requirements, equipment cost and energy consumption. Then, we utilized the non-linear programming problem to model the minimum SSU selection by taking the energy efficiency and real-timing into account. In view of the NP-hardness of the problem, we also developed a heuristic algorithm named SSUS to efficiently find the near-optimal solutions. The simulation results demonstrate that the proposed SSUS improves the energy efficiency while meeting the requirements both in end-to-end delay and communication reliability.

## Figures and Tables

**Table 1:** Description of different types of SUs.

Terms	Definitions
CRUs	Equipped with CRs
NCRUs	Not equipped with CRs
OCRUs	CRs under the open status
CCRUs	CRs under the closed status
SSUs	CRs under the sensible status
NSSUs	CRs under the insensible status
